# Grazyna Jasienska, *The Fragile Wisdom*

**DOI:** 10.1093/emph/eot006

**Published:** 2013-04-10

**Authors:** Maxine Weinstein

**Affiliations:** Center for Population and Health Graduate School of Arts and Sciences Georgetown University *E-mail: weinstma@georgetown.edu*

Do unto others; first do no harm; ask questions. And in this ambitious volume, Grazyna Jasienska poses a host of thoughtful questions, enough to keep a stable of researchers going for many lifetimes. The central query is whether an evolutionary perspective can help us understand the relationship between reproductive function and health. Although she is circumspect about drawing inferences that might be prescriptive, the ultimate goal is to inquire whether we can see our way to identifying behaviors for improving health. No small potatoes here!

Indeed, although Jasienska primarily addresses female reproduction and health, that constraint still leaves us with a gargantuan task—a far-reaching inquiry across dimensions of time and populations. To understand just how extensive the scope of this work is, it is instructive to consider some of the questions that need to be answered—or at least investigated—in order to get at the overarching theme, and then try to understand the kind of evidence that is required.

It seems to me there are three essential elements. The first task is ‘simply’ to document the range and variability in fundamental reproductive parameters: the underlying steroids and gonadotropins and cycle characteristics, for example. The second task is to identify and document the correlates of those factors and whether and how they vary (again, for example) by age, ecological setting, social practices, ancestry (or inheritance) or lifestyles (exercise, nutrition, exposure to challenge of various kinds, etc.). Third, we need to think about what health outcomes might meaningfully be affected by reproductive function and history, and document those linkages.

Jasienska takes an evolutionary perspective as her starting point in posing these questions. It is a provocative and creative approach to identifying possible pathways for research; it is also an approach that places a heavy burden on finding conclusive—or even suggestive—evidence. Most populations for which we have good data—although there are some exceptions which Jasienska cites—are also reasonably well nourished, limit childbearing, and engage in activities that are likely to be less strenuous than our forebears. Can we assume, for example, that data on current hunter–gatherer populations accurately reflect the status of ancestral populations? Or again, can we extrapolate from highly selected populations to make more general inferences about adaptive mechanisms?

As an example, Jasienska proposes polymorphisms in steroid-producing genes as an intriguing example of how this evolutionary perspective leads to hypotheses linking reproductive function and health. The idea is that if selection pressures were high enough, differences in the use of phytoestrogens in food would result in corresponding differences in the frequency of alleles that play a part in steroid production. According to this hypothesis, Jasienska argues that we would expect to find the highest frequencies of the A2 allele of CYP17, which is associated with high estrogen levels, among women of Asian descent where consumption of phytoestrogens is high thereby suppressing endogenous estrogen production in premenopausal women, and the lowest among women of African or European descent where it is low. And indeed, as she documents, this result is what we find.

But if our ultimate interest is in understanding links between reproductive parameters and health, what does this genotypic information imply about the prevalence of cancers thought to be linked to steroid exposure? To help answer this question, we can use Jasienska’s summary of extant findings from various studies across populations of varied ethnic descent, of genotype frequency categorized by disease outcome.

Here, I will focus on breast cancer for which the association between levels of endogenous steroid hormones and the disease is well documented. I use Jasienska’s excellent summary of data to extract only the entries for which she provides both cases and controls. Admittedly, she presented these data by way of estimating the distribution of genotypes, but we can exploit it for other purposes as well, in this case, as a quick check on whether the genotype modifies risk.

Overall, data on breast cancer from studies with both cases and controls show that 19% of the women are A2/A2, but only 16% of breast cancer cases are A2/A2 (*P* > 0.05). One might expect to find risk to be most greatly affected by genotype among women of Japanese and ‘Other East Asian’ descent because of their additional exposure to phytoestrogens. However, the relevant values for Japanese women are 16% A2/A2 among controls and 14% among cases (*P* > 0.05) and for ‘Other East Asian’, the values are in the opposite direction: 33% (controls) and 37% (cases).

As a non-expert in this area, I recognize the need to tread very cautiously here, but a quick search of the literature suggests that it is not clear whether there is a link between CYP17 genotype and breast cancer. That is, studies on this issue seem consistently inconsistent.

As a demographer, I would probably approach a comparative analysis of breast cancer from a different direction. For instance, the patterns of age-specific incidence of breast cancer rates for the USA, Japan and Korea differ dramatically [1]. In the USA, incidence rises rapidly premenopausally and continues to rise postmenopausally until flattening off in women’s 70s. By contrast, rates level off in Japan from ages 40 to 69 years, after which they decline, whereas in Korea breast cancer declines steadily after age 40 years. What could explain this picture? A couple of thoughts come to mind, but the distribution of genotype frequencies would be low on the list: the requisite effects would simply be too large. Ignoring data quality issues, and as a former student of Norm Ryder, I’d certainly start by examining cohort trends, looking, for example at incidence rates at younger ages for the older cohorts. What were the rates that we now see at age 50 (say)? Were they substantially lower at age 20 years than the current rates at age 20? Then, I might look at behavioral differences at the peri- and post-menopausal ages. Are there differences in the extent to which these populations use hormone replacement therapies? And of course, one would consider selective mortality as well as differential screening and diagnosis practices. One very obvious potential explanation comes to mind: dietary habits and cohort changes in those practices. Has the ‘Westernization’ of diet in Japan and Korea resulted in increasing rates for younger women in those countries? Undoubtedly, an expert on breast cancer would be able to propose other explanatory factors as well.

Does a demographic perspective tell us more than an evolutionary approach? My hope would be that the two are complementary. Perhaps the evolutionary perspective is a cause for greater optimism if we are hopeful about the promise of personalized or predictive medicine, gene therapies, or prescribing behavior change on the basis of knowing an individual’s genome. I remain skeptical.

These kinds of concerns, however, do not detract from a general assessment of this volume. Indeed, they serve to underscore its interest to a wide range of potential readers. The book is provocative, raises good questions, and could direct a generation of researchers in new directions.

## References

[1] Jung YS, Na KY, Kim KS (2011). Nation-wide Korean breast cancer data from 2008 using the breast cancer registration program. J Breast Cancer.

